# Supplementary material for: Endothelial glycocalyx and cardio-renal risk factors in type 1 diabetes
Source: PLoS One. 2021 Jul 30;16(7):e0254859. doi: 10.1371/journal.pone.0254859 (PMC8323905; doi:10.1371/journal.pone.0254859)
Supplement: S1 Questionnaire — (DOCX) [file pone.0254859.s001.docx]

**Participant number: _____________**

QUESTIONNAIRE REGARDING

**PROTON**

*“PeRsOnalising Treatment Of diabetic Nephropathy:*

*From albuminuria to multidimensional characterisation of diabetic nephropathy”*

All answered questions and all survey results remain

treated in strict confidence as the rules of medical professional secrecy

prescribes. Only healthcare professionals will see

the survey results and the information you provide. As soon as

the investigation is completed, all personal data will be destroyed.

Please answer the questions asked by ticking the answer

you yourself think is the most right. Some questions are answered by

stating some numbers or a few words.

Do not answer if you do not understand a question.

Bring the questionnaire the day of your appointment, then we can

together fill in the questions you have been in doubt about.

Name : .

Date : .

| Diseases of the gastrointestinal tract |
| --- |

Has a doctor ever told you that you had / have had:

**1.** Gluten allergy (Celiac intolerance)

(choose one)?

- Yes
- No
- Don´t know

If yes, enter the year of diagnosis:

_____­­­­­­____________________________

**2.** Milk allergy (Lactose intolerance)

(choose one)?

- Yes
- No
- Don´t know

If yes, enter the year of diagnosis:

_____­­­­­­____________________________

**3.** Gastrointestinal cancer (choose one)?

- Yes
- No
- Don´t know

If yes, enter the year of diagnosis:

_____­­­­­­___________________________

If so, where was the cancer located (choose one)?

- In the colon and/or rectum
- In the stomach
- In the esophagus
- In the pancreas
- In the liver and bile ducts
- Don´t know

**4.** Pouches on the colon (diverticula) (select one)?

- Yes
- No
- Don´t know

If yes, enter the year of diagnosis:

_________________________________

**5.** Gastric ulcer and/or duodenal ulcer (choose one)?

- Yes
- No
- Don´t know

If yes, enter the year of diagnosis:

_____­­­­­­____________________________

If so, have you received treatment to remove ulcers/ulcers on the duodenum in the form of antibiotics and antacids (eradication treatment - often for 14 days)?

- Yes
- No
- Don´t know

**6.** Heartburn and / or acid reflux (choose one)?

- Yes
- No
- Don´t know

If yes, enter the year of diagnosis:

_____­­­­­­____________________________

**7.** Hepatitis (choose one)?

- Yes
- No
- Don´t know

If yes, enter the year of diagnosis:

__________________________________

**8.** Other liver disease (choose one)?

- Yes
- No
- Don´t know

If yes, enter the year of diagnosis:

_____­­­­­­____________________________

**9.** Crohn's disease or ulcerative colitis (inflammatory bowel disease) (choose one)?

- Yes
- No
- Don´t know

If yes, enter the year of diagnosis:

_____­­­­­­____________________________

**10.** Irritable bowel syndrome (choose one)?

**13.** Have you ever had surgery in the gastrointestinal tract (choose one)?

- Yes
- No
- Don´t know

If yes, tick the appropriate boxes:

- Removed appendix
- Removed gallstones
- Yes
- No
- Don´t know

If yes, enter the year of diagnosis:

_____­­­­­­____________________________

**11.** Have you had a stomach infection within the last 3 months, e.g. Roskilde disease (choose one)?

- Yes
- No
- Don´t know

If yes, enter the month of diagnosis:

- Colon or small intestine
- Gastric by-pass or banding
- Removed intestinal polyps
- Ulcer
- Other, describe:

________________________________

________________________________

________________________________

________________________________

________________________________

If so, when was the operation performed?

________________________________

_________________________________

**12.** Inflammation of the pancreas (choose one)?

- Yes
- No
- Don´t know

If yes, enter the year of diagnosis:

_________________________________

| Stool pattern |
| --- |

**1.** What is your average bowel movement rate (choose one)?

- Twice daily or more often
- Once daily
- Once every other day
- Less often than once every other day
- Don´t know

**2.** Is your stool regular (choose one)?

- Yes
- No
- Don´t know

**3.** Do you suffer from bloating / flatulence (choose one)?

- Yes
- No
- Don´t know

**4.** What type of stool do you have most often according to the below Bristol scale for stool types (choose one)?

BRISTOL SCALE for stool types

|  | - Type 1 | Separate, hard lumps, like nuts |
| --- | --- | --- |
|  | - Type 2 | Like a sausage, though in lumps |
|  | - Type 3 | Like a sausage/snake, smooth and soft |
|  | - Type 4 | Like a sausage, but with cracks in the surface |
|  | - Type 5 | Soft blobs with clear edges |
|  | - Type 6 | Soft stools with uneven edges |
|  | - Type 7 | Aqueous consistency, no solid parts |

| Contact with animals |
| --- |

**1.** Are you in regular contact with animals (choose one)?

- Yes
- No

**If yes:**

If it is **at work**, please check which animals:

- Cat
- Dog
- Farm animals
- Rodent
- Others, please indicate which:

________________________________________________________________________ _______________

If it's **at home** (I keep animals), please check which animals:

- Cat
- Dog
- Farm animals
- Rodent
- Others, please indicate which:

________________________________________________________________________ _______________

If you are in contact with animals elsewhere than at home, please indicate where:

_______________________________________________________________________________________

_______________________________________________________________________________________

__________________________________________________________________________________ __

| \| Tobacco and smoking habits \| \| --- \| | | | |
| --- | --- | --- | --- | --- |
| **1.** Do you smoke? | | | |
| - Yes, daily | |  | |
| - Occasionally (less than 1 cigarette / cigar / cerut / pipe daily) | |  | |
| - No | |  | |
|  | | | |
| **2** If you do not smoke now, have you smoked before? | | | |
| - Yes, daily | |  | |
| - Occasionally (less than 1 cigarette / cigar / cerut / pipe daily) | |  | |
| - No, never | |  | |
|  | | | |
| **3** If you quit smoking, when did you quit? | | year | |
|  | | | |
| **4** How much do you smoke, or did you smoke, on average per day? | | | |
| - Cigarettes with filters daily | | quantity | |
| - Cigarettes without filter daily | | quantity | |
| - Ceruts daily | | quantity | |
| - Cigars daily | | quantity | |
| - Pipe tobacco (grams) daily | | quantity | |
| \| Alcohol \| \| --- \| | | | |
| **1.** Do you drink alcohol (beer, wine or spirits)? | yes | | no |
| - If yes, number of items/week | quantity | | |
|  | | | |
| **1.1** Do you drink: |  | | |
| - Beer |  | | |
| - Wine |  | | |
| - Spirits |  | | |
|  | | | |
| \| Physical activity \| \| --- \|   The following is about how physically active you are both at work and in your spare time.  By physical activity is meant all activities where you get to move your muscles and use your strength. So you don't just think about exercise, sports or the like.  **The questions below deal with DAILY activities:**   1. In your spare time, how many hours and minutes a day do you spend approximately watching TV, sitting down and relaxing, reading and listening to music or the like?   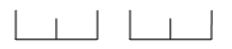  Hours Minutes   1. How long do you sleep approximately on a regular weekday? (including a nap)   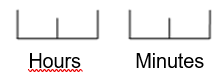   1. Are you at working or studying?  - Yes (Go to question 4) - No (Go to question 8)   In your work (or during studying), how many hours and minutes a day do you typically spend on:   1. Sedentary work?   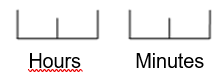   1. Standing or walking work?   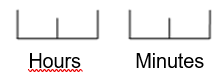     1. Hard physical work? (Eg heavy lifting or stair climbing)   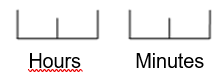     1. How many hours and minutes do you spend daily on cycling or walking in connection with transport to and from work/education?   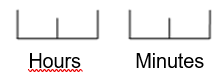 | | | |
|  | | | |

**The following questions concerns WEEKLY activities:**

1. In your spare time, how many hours and minutes do you spend per week on light physical activity such as walks, easy cleaning, sweeping and working in the garden or light strenuous exercise such as yoga, bowling or similar?


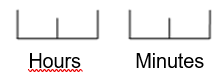


1. In your spare time, how many hours and minutes do you spend a week on gardening, carrying things up the stairs or moderately strenuous sports such as gymnastics, swimming, cycling, strength training or the like? (Do not include transport to and from work)


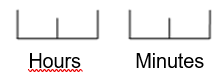


1. In your spare time, how many hours and minutes a week do you spend on strenuous sports and exercise such as running, jogging, football, tennis, aerobics or the like? (Do not include transport to and from work)


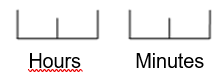


1. All in all, how do you assess your physical shape?

- Really good
- Good
- Roughly
- Less good
- Bad
