# Supplementary material for: Endothelial glycocalyx and cardio-renal risk factors in type 1 diabetes
Source: PLoS One. 2021 Jul 30;16(7):e0254859. doi: 10.1371/journal.pone.0254859 (PMC8323905; doi:10.1371/journal.pone.0254859)
Supplement: S2 Questionnaire — (DOCX) [file pone.0254859.s002.docx]

**Deltager nummer: _______________**

SPØRGESKEMA VEDRØRENDE

**PROTON**

*“PeRsOnalising Treatment Of diabetic Nephropathy:*

*From albuminuria to multidimensional characterisation of diabetic nephropathy”*

Alle besvarede spørgsmål og alle undersøgelsesresultater bliver behandlet strengt fortroligt som reglerne om lægelig tavshedspligt foreskriver. Kun sundhedsfagligt personale vil se undersøgelsesresultaterne og de oplysninger, du giver. Så snart undersøgelsen er afsluttet, bliver alle personrelaterede data tilintetgjort.

Besvar venligst de stillede spørgsmål ved at sætte kryds ud for det svar, du selv mener, er det mest rigtige. Enkelte spørgsmål besvares ved at anføre nogle tal eller nogle få ord.

Undlad at svare, hvis du ikke forstår et spørgsmål.

Medbring spørgeskemaet den dag du har tid til undersøgelse, så kan vi sammen udfylde de spørgsmål, du har været i tvivl om.

Navn : .

Dato : .

| Sygdomme i mavetarmregionen |
| --- |

Har en læge nogensinde fortalt dig, at du havde/har haft:

**1.** Glutenallergi (Cøliaki-intolerance)

(vælg én)?

- Ja
- Nej
- Ved ikke

Hvis ja, angiv årstal for diagnose:

_____­­­­­­____________________________

**2.** Mælkeallergi (Laktose-intolerance)

(vælg én)?

- Ja
- Nej
- Ved ikke

Hvis ja, angiv årstal for diagnose:

_____­­­­­­____________________________

**3.** Kræft i fordøjelseskanalen (vælg én)?

- Ja
- Nej
- Ved ikke

Hvis ja, angiv årstal for diagnose:

_____­­­­­­____________________________

Hvis ja, hvor sad kræften (vælg én)?

- I tyk- og/eller endetarmen
- I mavesækken
- I spiserøret
- I bugspytkirtlen
- I lever-og galdeveje
- Ved ikke

**4.** Udposninger på tyktarmen (divertikler) (vælg én)?

- Ja
- Nej
- Ved ikke

Hvis ja, angiv årstal for diagnose:

_____­­­­­­____________________________

**5.** Mavesår og/eller sår på tolvfingertarmen (vælg én)?

- Ja
- Nej
- Ved ikke

Hvis ja, årstal for diagnose:

_____­­­­­­____________________________

Hvis ja, har du modtaget behandling for at fjerne mavesår/sår på tolvfingertarmen i form af antibiotika og syrenedsættende behandling (eradikationsbehandling - ofte i 14 dage)?

- Ja
- Nej
- Ved ikke

**6.** Halsbrand og/eller sure opstød (vælg én)?

- Ja
- Nej
- Ved ikke

Hvis ja, angiv årstal for diagnose:

_____­­­­­­____________________________

**7.** Leverbetændelse (vælg én)?

- Ja
- Nej
- Ved ikke

Hvis ja, angiv årstal for diagnose:

__________________________________

**8.** Anden leversygdom (vælg én)?

- Ja
- Nej
- Ved ikke

Hvis ja, angiv årstal for diagnose:

_____­­­­­­____________________________

**9.** Crohns sygdom eller colitis ulcerosa (inflammatorisk tarmsygdom) (vælg én)?

- Ja
- Nej
- Ved ikke

Hvis ja, angiv årstal for diagnose:

_____­­­­­­____________________________

**10.** Irritabel tyktarm (vælg én)?

**13.** Har du nogensinde været opereret i mave- eller tarmregionen (vælg én)?

- Ja
- Nej
- Ved ikke

Hvis ja, sæt kryds i de relevante bokse:

- Fjernet blindtarm
- Fjernet galdesten
- Ja
- Nej
- Ved ikke

Hvis ja, angiv årstal for diagnose:

_____­­­­­­____________________________

**11.** Har du haft maveinfektion indenfor de sidste 3 måneder, fx. Roskildesyge (vælg én)?

- Ja
- Nej
- Ved ikke

Hvis ja, angiv måned for diagnose:

_________________________________

- Tyktarm eller tyndtarm
- Gastric by-pass eller banding
- Fjernet polypper i tarm
- Mavesår
- Andet, beskriv:

________________________________

________________________________

________________________________

________________________________

________________________________

Hvis ja, hvornår blev du opereret?

________________________________

**12.** Betændelse i bugspytkirtlen (pancreas) (vælg én)?

- Ja
- Nej
- Ved ikke

Hvis ja, angiv årstal for diagnose:

_________________________________

| Afføringsmønster |
| --- |

**1.** Hvad er din gennemsnitlige afføringshyppighed (vælg én)?

- 2 gange dagligt eller oftere
- 1 gang dagligt
- 1 gang hver anden dag
- Sjældnere end én gang hver anden dag
- Ved ikke

**2.** Er din afføring regelmæssig (vælg én)?

- Ja
- Nej
- Ved ikke

**3.** Lider du af oppustethed/flatulens (vælg én)?

- Ja
- Nej
- Ved ikke

**4.** Hvilken type afføring har du oftest i henhold til nedenstående Bristol-skala for afføringstyper (vælg én)?

BRISTOL SKALAEN for afføringstyper

|  | - Type 1 | Separate, hårde klumper, som nødder |
| --- | --- | --- |
|  | - Type 2 | Som en pølse, dog i klumper |
|  | - Type 3 | Som en pølse/slange, jævn og blød |
|  | - Type 4 | Som en pølse, men med revner i overfladen |
|  | - Type 5 | Bløde klatter med klare kanter |
|  | - Type 6 | Blød afføring med ujævne kanter |
|  | - Type 7 | Vandet, ingen faste dele |

| Kontakt til dyr |
| --- |

**1.** Er du regelmæssigt i kontakt med dyr (vælg én)?

- Ja
- Nej

**Hvis ja:**

Hvis det er **på arbejdet**, afkryds venligst hvilke dyr:

- Kat
- Hund
- Bondegårdsdyr
- Gnaver
- Andre, angiv venligst hvilke:

________________________________________________________________________ _______________

Hvis det er **i hjemmet (jeg holder dyr)**, afkryds venligst hvilke dyr:

- Kat
- Hund
- Bondegårdsdyr
- Gnaver
- Andre, angiv venligst hvilke:

________________________________________________________________________ _______________

Hvis du er i kontakt med dyr **andre** steder end i hjemmet, angiv venligst hvor:

_______________________________________________________________________________________

_______________________________________________________________________________________

__________________________________________________________________________________ __

| \| Tobak og rygevaner \| \| --- \| | | | |
| --- | --- | --- | --- | --- |
| **1.** Ryger du? | | | |
| - Ja, dagligt | |  | |
| - Af og til (mindre end 1 cigaret/cigar/cerut/pibe dagligt) | |  | |
| - Nej | |  | |
|  | | | |
| **2** Ryger du ikke nu, har du da tidligere røget? | | | |
| - Ja, dagligt | |  | |
| - Af og til (mindre end 1 cigaret/cigar/cerut/pibe dagligt) | |  | |
| - Nej, aldrig | |  | |
|  | | | |
| **3** Hvis du er ophørt med at ryge, hvornår holdt du op? | | årstal | |
|  | | | |
| **4** Hvor meget ryger du, eller røg du, gennemsnitlig om dagen? | | | |
| - Cigaretter med filter dagligt | | antal | |
| - Cigaretter uden filter dagligt | | antal | |
| - Cerutter dagligt | | antal | |
| - Cigarer dagligt | | antal | |
| - Pibetobak (gram) dagligt | | antal | |
| \| Alkohol \| \| --- \| | | | |
| **1.** Drikker du alkohol (øl, vin eller spiritus)? | ja | | nej |
| - Hvis ja, antal genstande/uge | antal | | |
|  | | | |
| **1.1** Drikker du: |  | | |
| - Øl |  | | |
| - Vin |  | | |
| - Spiritus |  | | |
|  | | | |
| \| Fysisk aktivitet \| \| --- \|   Det følgende handler om, hvor meget du bevæger dig såvel på arbejde som i fritiden.  Med bevægelse menes her alle aktiviteter, hvor du får rørt dine muskler og bruger dine kræfter. Der tænkes altså ikke kun på motion, idræt eller lignende.  **Nedenstående spørgsmål omhandler DAGLIGE aktiviteter:**   1. I din fritid, hvor mange timer og minutter om dagen bruger du ca. på at se TV, sidde ned og slappe af, læse og lytte til musik eller lignende?   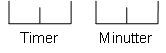   1. Hvor lang tid sover du ca. på et almindeligt hverdagsdøgn? (Medtag middagslur)   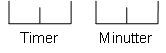   1. Er du i arbejde eller under uddannelse  - Ja (Gå til spørgsmål 4) - Nej (Gå til spørgsmål 8)   I dit arbejde (eller under uddannelse), hvor mange timer og minutter om dagen bruger du typisk på:   1. Stillesiddende arbejde?   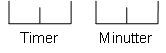   1. Stående eller gående arbejde?   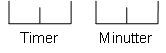     1. Hårdt fysisk arbejde? (F.eks. tunge løft eller trappegang)   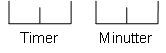     1. Hvor mange timer og minutter bruger du dagligt på cykling eller gang i forbindelse med transport til og fra arbejde/uddannelse?   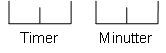 | | | |
|  | | | |

**Nedenstående spørgsmål omhandler UGENTLIGE aktiviteter:**

1. I din fritid, hvor mange timer og minutter bruger du om ugen på let fysisk aktivitet som f.eks. gåture, let rengøring, feje og rive i haven eller let anstrengende motion som f.eks. yoga, bowling eller lignende?


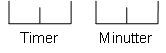


1. I din fritid, hvor mange timer og minutter bruger du om ugen på havearbejde, bære ting op af trappen eller moderat anstrengende sport som f.eks. gymnastik, svømning, cykling, styrketræning eller lignende? (Medtag ikke transport til og fra arbejde)


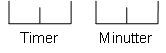


1. I din fritid, hvor mange timer og minutter om ugen bruger du på anstrengende sport og motion som f.eks. løb, jogging, fodbold, tennis, aerobic eller lignende? (Medtag ikke transport til og fra arbejde)


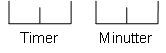


1. Alt i alt hvordan vurderer du din fysiske form?

- Virkelig god
- God
- Nogenlunde
- Mindre god
- Dårlig
